# Flexible navigation response in common cuckoos *Cuculus canorus* displaced experimentally during migration

**DOI:** 10.1038/srep16402

**Published:** 2015-11-09

**Authors:** Mikkel Willemoes, Julio Blas, Martin Wikelski, Kasper Thorup

**Affiliations:** 1 Center for Macroecology, Evolution and Climate; Natural History Museum of Denmark; University of Copenhagen; Universitetsparken 15; 2100 København Ø; Denmark; 2 Department of Conservation Biology, Estación Biológica de Doñana, CSIC, c/Americo Vespucio, 41092 Sevilla, Spain; 3 Max Planck Institute for Ornithology, Department of Migration and Immuno-ecology, Am Obstberg 1, D-78315 Radolfzell, Germany; 4 University of Konstanz, Department of Biology, Universitätsstrasse, D-78457 Konstanz, Germany

## Abstract

Migrating birds follow innate species-specific migration programs capable of guiding them along complex spatio-temporal routes, which may include several separate staging areas. Indeed, migration routes of common cuckoos *Cuculus canorus* show little variation between individuals; yet, satellite tracks of 11 experimentally displaced adults revealed an unexpected flexibility in individual navigation responses. The birds compensated for the translocation to unfamiliar areas by travelling toward population-specific staging areas, demonstrating true navigation capabilities. Individual responses varied from travelling toward the first stopover in northern Europe to flying toward the Central-African winter grounds, the latter including several stopovers in unfamiliar areas. Apparently, the cuckoos possess spatial knowledge far beyond their population-specific flyway scale, and make individual decisions likely based on an assessment of perceived gain and cost of alternative route options.

The complex innate spatio-temporal migration programs, capable of guiding migrant species thousands of kilometers, represents an evolutionary trade-off between species-specific resource needs and movement-related risks, resulting in varying migratory patterns across species and populations^1^,^2^. The navigational basis of this program is still an unsolved mystery despite decades of research^3^. To investigate the navigational capabilities in birds, experimental displacement is a common practice^4^. Experienced songbird migrants can perform true navigation involving the use of a map sense to identify the position of the current location in relation to a goal, enabling them to compensate for a displacement, even outside familiar areas^5^. This has been documented using various methods based on migration directions of displaced birds, such as ring recoveries^6^,^7^, radio tracking^8^,^9^, and orientation cages^10^,^11^,^12^. However, the actual movement paths are critical to understand the processes of navigation^3^ and only recent development of remote tracking technologies has made this now possible^13^.

We investigate the navigational capabilities in experienced individuals of a solitary, nocturnal migrant by experimentally displacing adult common cuckoos, a long-distance migrant with one of the most complex migrations known, using seven different staging sites between each breeding season^14^. Immediately before the usual start of post-breeding migration, we displaced 12 birds from breeding grounds in northern Europe (Denmark) more than 2500 km to south-westernmost Europe (Spain), over 1000 km from the route normally followed by cuckoos breeding in Denmark (Fig. 1A).

Shorter-distance (~100 km) directional responses in white-crowned sparrows suggest that experienced birds choose to travel directly toward their winter grounds after a long-distance displacement during the autumn migration^8^. Similarly, the single recovery during spring migration of a white-crowned sparrow displaced during winter suggests travelling toward the breeding grounds^7^. In both cases, the birds seem to travel toward the end goal for that season. In general, we expect a fixed programmed response to a displacement based on the importance of each stopover site in combination with the seasonal progress and displacement distance and direction. For a species with many intermediate goals (staging sites) like the common cuckoo, the importance of each site is not known and likely changes during the migration period. Thus, individual responses to displacement will provide major insights into this system.

## Results

**Figure 1:**
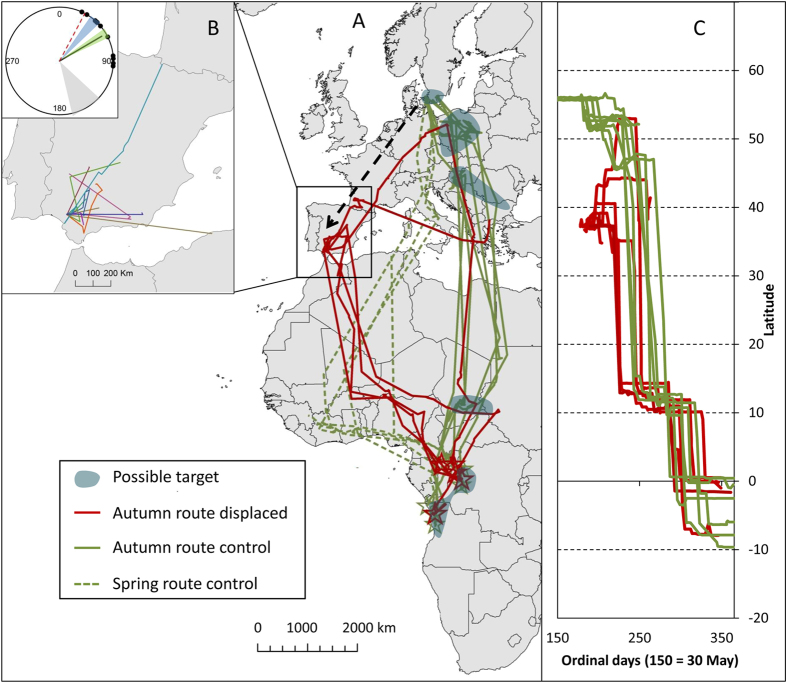
Tracking of displaced common cuckoos in time and space. (**A**) Autumn migration routes of the six adult common cuckoos whose navigation was successfully monitored (red tracks) after being displaced from Denmark to Spain (arrow) compared to the migrations of eight adult non-displaced common cuckoos from Denmark and southern Sweden (green tracks: autumn vs spring routes indicated as solid vs dotted lines respectively). Stars indicate final wintering destination and shaded areas indicate population-specific autumn staging areas considered as possible navigation targets. (**B**) Initial movements of 11 adult common cuckoos within the 15 days following displacement including endpoint directions and mean direction (black dots and solid black line in circle diagram) compared to direction towards origin (red dotted line), first and second staging areas (blue and green triangles) of control birds and first post-breeding migration departure direction (grey triangle) of control birds. (**C**) Timing of autumn migration of displaced birds (red lines) compared to control birds (green lines). Maps are created in ArcMap 10.1^32^ (Mercator projection).

The initial response to displacement was movement toward east-northeast (direction from release site to location 15 days after displacement: mean = 060°, r = 0.91, p < 0.0001, 95% confidence interval = [046°, 074°]; Fig. 1B and Supplementary Table 1). This direction was significantly different from the south-easterly direction (mean = 144°, r = 0.95) of the first movements of the non-displaced cuckoos (n = 8) from southern Scandinavia^14^ (Watson-Williams test: F_1,17_ = 64.8, P < 0.0001). The movements compensated for the displacement and were directed toward the post-breeding migration route normally followed by non-displaced birds. The pattern was similar for endpoint directions after 100 and 200 km (Supplementary Table 1 and 2). On average, the flights were oriented toward the nearest part of this route, similar to the direction toward the second European autumn staging site (055°–064°) but differing from both the direction to the capture location (028°) and to the first staging site (036°–046°) (P < 0.05, confidence interval test) used by the non-displaced birds. The directions of initial steps (within 15 days and longer than 5 km) revealed no difference among individuals (Watson-Williams test: F_9,40_ = 0.33, p = 0.96).

Six displaced birds reached the migration route of the control birds after travelling 43–147 days (the remaining six birds stopped transmitting after 0–38 days, see also Supplementary Table 3). All homed to staging areas normally used by this population during autumn migration but individuals travelled to areas with large geographical separation (Fig. 1A and Supplementary Video). Travel directions from release to the route of control birds differed among individuals (Watson-Williams test: F_5,155_ = 8.1, p < 0.0001).

Two birds continued their initial movements in eastern and north-eastern directions until they reached the migration corridor of the non-displaced birds. One moved to the first staging site in North Central Europe and continued on a route similar to the non-displaced birds. The other bird moved toward the staging area in Southeast Europe, but transmission stopped just before arrival. The remaining four all left Spain and travelled through North Africa to cross the Sahara desert in a south to south-eastern direction, similar to the direction chosen by non-displaced conspecifics when crossing the Sahara southwards (169.3° vs 174.4° respectively). After crossing the desert, these four birds visited staging sites on latitudes corresponding to the Sahel stop over area (12.4°N vs 11.8°N, Fig. 1C) used by non-displaced birds, although on much more westerly longitudes (6.7°E vs 19.2°E). During their stay in Sahel, the birds moved east and one continued to the staging area in eastern Sahel, whereas the remaining three migrated to the winter area.

## Discussion

The displaced cuckoos demonstrated an ability to navigate back to their normal migration route. The displacement location and routes travelled were most likely unfamiliar thus indicating the use of true navigation, with stopovers used by non-displaced birds seemingly acting as goal areas. Despite the initial response among individuals being similar, the variation in the later part of the birds’ routes shows a highly flexible overall response to the displacement.

The similar initial north-eastern orientation response could indicate navigation toward the temporally appropriate areas. The later variation in location of individual goal area could result from differences in timing, following a moving goal area^15^. However, this seems unlikely because the birds that migrated directly to the Sahel from Spain, reached the Sahel stopovers one month earlier than non-displaced conspecifics (average arrival date to Sahel was 19 August for displaced birds versus 20 September for non-displaced birds, Fig. 1C) when the temporally appropriate location was still in Europe. Thus, their strategy for compensating for displacements appears to be highly flexible and adjusted individually. In order to select an individual beneficial strategy, birds should be capable of balancing perceived gains and risks of several different response scenarios. Such a task would require knowledge of the current location in relation to all of the possible goals as well as distances to each of the goals and our findings illustrate the complexity of the migratory orientation.

The relatively direct routes followed by displaced birds toward the staging areas used by non-displaced birds, suggest an important goal-oriented strategy during migration, and the observed flexibility in response to displacement is unlikely to be based on variation in the inherited spatio-temporal migration programme alone. Inter-individual differences in the chosen routes could result from differences in physiological condition or experience during prior migrations. Physiological condition has been shown to affect migration decisions^16^,^17^ but little is known about the effect of experience within adults. Low individual route fidelity of adults in some species^18^,^19^, suggests that route flexibility is high despite experience from previous migrations. Black kites *Milvus migrans* migrating from Spain to West Africa steadily increased route repeatability up until the age of seven years when it levelled out^20^. It is therefore possible, that differences in age even within adults can lead to different levels of route fidelity. Additionally, personality traits could potentially have an effect as such traits have also been documented to affect migration decisions^21^.

The initial compensatory reaction suggests the use of true navigation because the release site is well outside the normal migration route of this population. The spring migration routes followed did not differ between displaced and non-displaced birds except for one displaced bird that crossed the Mediterranean Sea between Morocco and Spain instead of between Tunisia and Italy (Supplementary Figure 1). It is unknown whether this individual normally uses such an aberrant spring route or possibly learned it from being displaced to southern Spain. Temporally, the displaced birds departed West Africa and arrived in Europe earlier than the non-displaced birds (Supplementary Figure 1), however the tracks are from different years and the cuckoos in general arrived later in Denmark in 2011 (non-displaced birds) than in 2012 and 2014 (displaced birds, Supplementary Figure 2).

Overall, it appears that crossing familiar terrain was not needed for navigation. Four of the six birds ultimately returning to normal migration route, crossed the Sahara directly after leaving Spain in a direction similar to the direction used by control birds and only navigated toward their normal route when reaching the familiar areas of the spring (pre-breeding northbound) migration route. However, the birds stopped over in and navigated across the Sahel in latitudes 800–1100 km north of the spring routes followed by non-displaced birds between Central Africa and West Africa (Fig. 1A). First during the last 800 km before reaching the Central African wintering area did they follow routes similar to those followed in spring and only one individual navigated to the stopover in Sahel used by non-displaced birds after crossing familiar areas.

The navigational capability displayed by the adult cuckoos in this study indicates the existence of an extensive map sense reaching far outside the area normally traversed by individual birds. If the birds are relying on a gradient map^22^, the direct homing would require at least two gradient axes. The sensory basis for such a map is a subject of major interest although it has only been investigated in a few species^23^. A geomagnetic basis of the underlying map has been suggested for various organisms including spiny lobsters^24^, amphibians^25^, and sea-turtles^26^. However, a geomagnetic map would presumably not perform well in the area of release due to a relatively flat longitudinal gradient in both inclination and intensity^27^. A number of studies have suggested involvement of celestial cues as basis for navigation^11^. Olfactory deprivation has been shown to impair free-flying navigation in a displaced experienced migrant^9^. Thus, even at the large scale considered here, the use of olfactory cues for navigation cannot be ruled out. Better available tracking technologies in the future will hopefully enable us to solve the mystery^28^.

## Methods

### Tracking

This study was carried out in accordance with Guidelines to the use of wild birds in research of the Ornithological Council^29^. Animal work was approved by the Danish Nature Agency by permission to the Copenhagen Bird Ringing Centre (J.nr. SN 302–009). We captured 5 adult cuckoos in 2011 (4 males and 1 female) and 7 adult cuckoos in 2013 (all males) at breeding sites in Denmark (55.64–56.00°N, 12.25–12.59°E) and displaced them to a suitable habitat in southern Spain near Seville (37.21°N, –6.18°E), 2500 km to the southwest (221.5°). All birds were caught and displaced in the period 24 June–7 July, immediately before normal population departure (median departure date of eight tracked birds: 6 July^14^). The birds were kept in captivity on average 48 hours (range: 26–74) before the release in Spain. During this time they were offered water and mealworms ad libitum. They were transported in covered pet transport cages on a commercial airline from Copenhagen to Seville. The birds were ringed and equipped with a 5 g solar PTT-100s (Microwave Telemetry Inc.) satellite transmitter fitted to the birds as a back-pack using a body harness made from a 2 mm braided nylon string. In 2011, the birds were released in the morning after staying overnight in Spain. In 2013, they were released in the evening immediately after arrival due to different flight schedules. The transmitters were programmed on a 10 h/48 h on/off duty cycle. Geographical positions of the transmitters were obtained from ARGOS/CLS Service Argos^30^.

### Data analyses

Position estimates from ARGOS are assigned to a location quality class (3–0, A-B and Z; 3 has highest and Z lowest accuracy). After excluding all positions of class Z, we only used the highest quality position per transmission cycle unless the bird was in active movement, in which case all positions were included (following the procedure described in [14]). The movements of the 12 displaced birds were compared to the migration of eight non-displaced common cuckoos tagged in Denmark and southern Sweden (see [14] for a detailed description of this migration).

We investigated the first, initial as well as the full, longer term navigational responses to the displacement. The initial response was defined as movements from the release site to the location after 15 days, potentially allowing for five transmission cycles. For comparison, we also used the first location after travelling 100 and 200 km. The full response was defined as movements from the release site to the first location on the normal migration route.

We used a Rayleigh’s test to evaluate whether the direction to the end-point of initial responses were random. End-point directions of the initial responses were tested against specific directions (toward release location and stop over locations) using a confidence interval test, where the specific direction was considered different from the displaced group, if it was not included in the 95% confidence interval for the displaced group. The confidence interval was calculated as a bootstrap confidence interval around the mean direction in a von Misses distribution.

We tested for differences among individuals in response to displacement for both first and full response by comparing directions of individual steps longer than 5 km using a Watson-Williams test^31^. The initial response to displacement was tested by comparing the mean end-point directions of the first movement of displaced and non-displaced birds, respectively, using a Watson-Williams test.

Statistical tests were done in R 3.1.0^32^ using the package “circular”. Map representations were done in ArcMap 10.1^33^.

Data are deposited in Movebank (www.movebank.org). 

## Additional Information

**How to cite this article**: Willemoes, M. *et al.* Flexible navigation response in common cuckoos *Cuculus canorus* displaced experimentally during migration. *Sci. Rep.* **5**, 16402; doi: 10.1038/srep16402 (2015).

## Supplementary Material

Supplementary Information

Supplementary Video
