# Supplementary material for: Flexible navigation response in common cuckoos Cuculus canorus displaced experimentally during migration
Source: Sci Rep. 2015 Nov 9;5:16402. doi: 10.1038/srep16402 (PMC4637880; doi:10.1038/srep16402)
Supplement: Supplementary Information [file srep16402-s1.pdf]

Supplementary Online Material for:

"Flexible navigation response in common cuckoos displaced experimentally during migration"

Mikkel Willemoes, Julio Blas, Martin Wikelski and Kasper Thorup

Supplementary Table 1

Mean direction and vector length of the endpoints after either 15 days, 100km or 200km, for both displaced and control birds. P-value is the p-value of a Rayleigh's test of randomness for the directions within each group and P-value control is the p-value of a Watson Williams test between each group and the control birds after 15 days.

Initial movement	Mean direction	Vector length	P-value	P-value control
15 days	60	0.91	<0.0001	<0.0001
100 km	69	0.76	0.0006	0.0002
200 km	63	0.71	0.002	0.0002
Control 15 days	148	0.97	<0.0001	
Control 100 km	144	0.95	<0.0001	
Control 200 km	144	0.95	<0.0001	

Supplementary Table 2

Directional angles used in tests for randomness and when comparing displaced group to control group. Direction after 15 days or 100 and 200 km are directions from release site to first point after the criteria in question. Direction of the Sahara crossing is the direction from the last location north of 33°N to the first location south of 20°N.

Individual	Displaced	Direction 15 days	Direction 100 km	Direction 200 km	Direction Saharah crossing
126282	Yes	31	36	36	-
126281	Yes	43	138	50	-
126278	Yes	85	84	80	186
126279	Yes	24	25	25	-
126280	Yes	45	4	45	176
108930	Yes	92	38	42	173
108931	Yes	64	134	134	160
107386	Yes	42	93	93	168
36426	Yes	87	89	89	-
107388	Yes	50	50	292	-
107387	Yes	95	77	95	-
19150	No	135	139	139	169
49466	No	165	137	137	179
57372	No	158	144	144	162
57374	No	132	111	111	170
36328	No	156	168	168	184
36331	No	152	162	162	180
36332	No	160	159	159	-
36487	No	129	127	127	178

Supplemetary Table 3

Summary table of the tracking of 12 adult common cuckoos experimentally displaced from Denmark to southern Spain

**ID**	**Year**	**sex**	**Tracked days**	**Reached route**	**After days**	**In region**	**Max dist to release (km)**
107386	2011	F	385	Yes	105	SC Africa	5016
36426	2011	M	22	No	-	-	336
107388	2011	M	16	No	-	-	208
107387	2011	M	38	No	-	-	679
36426	2011	M	0	No	-	-	-
126282	2013	M	80	Yes	77	SE Europe	2604
126281	2013	M	21	No	-	-	433
126278	2013	M	573	Yes	43	NE Europe	4747
126279	2013	M	7	No	-	-	233
126280	2013	M	738	Yes	147	SC Africa	4867
108930	2013	M	321	Yes	121	SC Africa	4641
108931	2013	M	325	Yes	91	E Sahel	5437

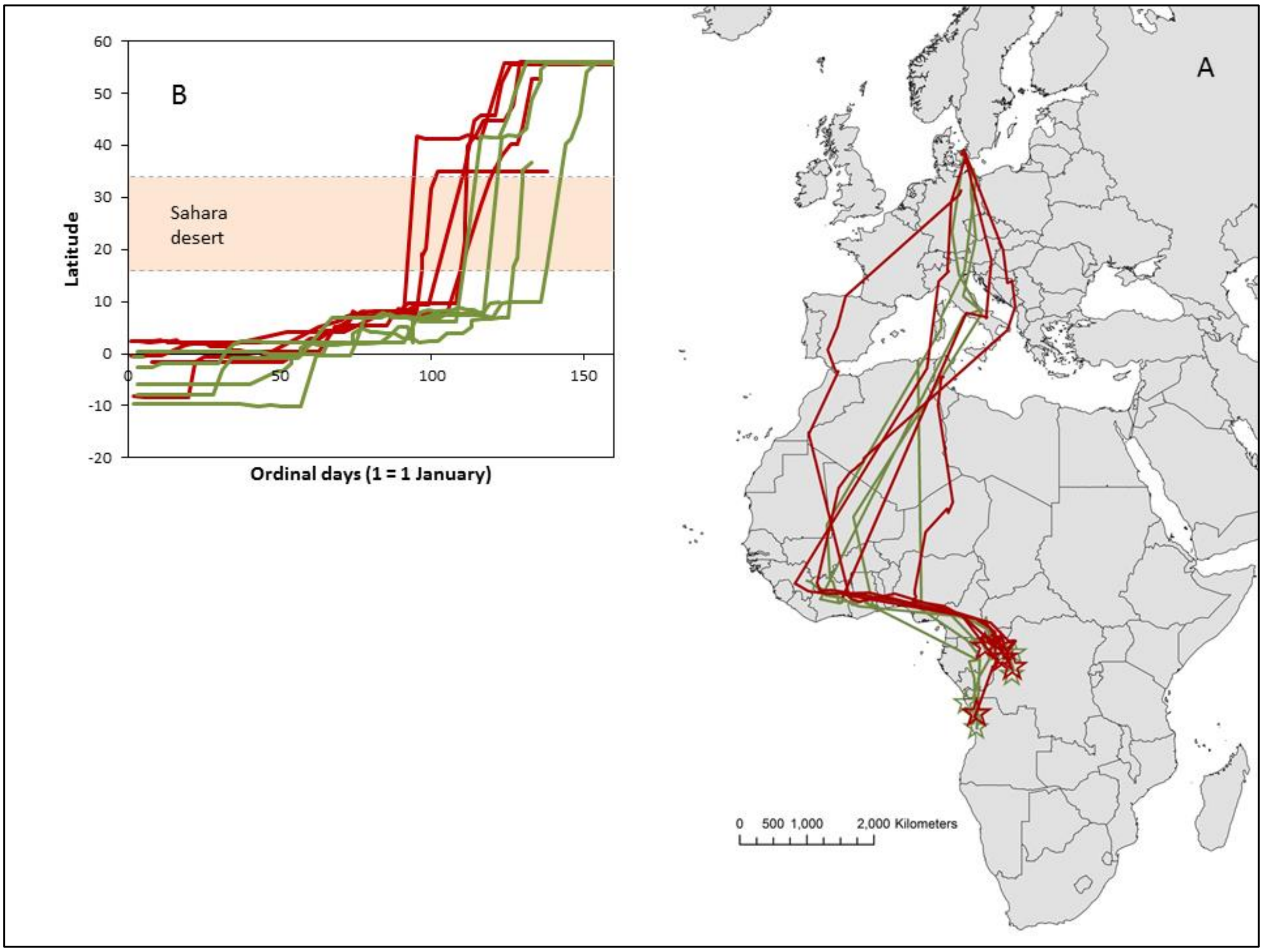

Supplementary Figure 1. A: routes followed during spring migration of non-displaced cuckoos (green) and cuckoos displaced the previous autumn (red) from the winter sites in Central Africa to Denmark. Note that one bird is flying through the Iberian Peninsula close to the release site. Whether this is due to an acquired knowledge of the area from the displacement treatment, or this bird did have previous experience with the Iberian Peninsula is unknown. Map is created using ArcMap 10.1 (Environmental Systems Research Institute, Redlands, CA), mercator projection. B: temporal progress of latitude during spring migration of non-displaced cuckoos (green) and cuckoos displaced the previous autumn (red).

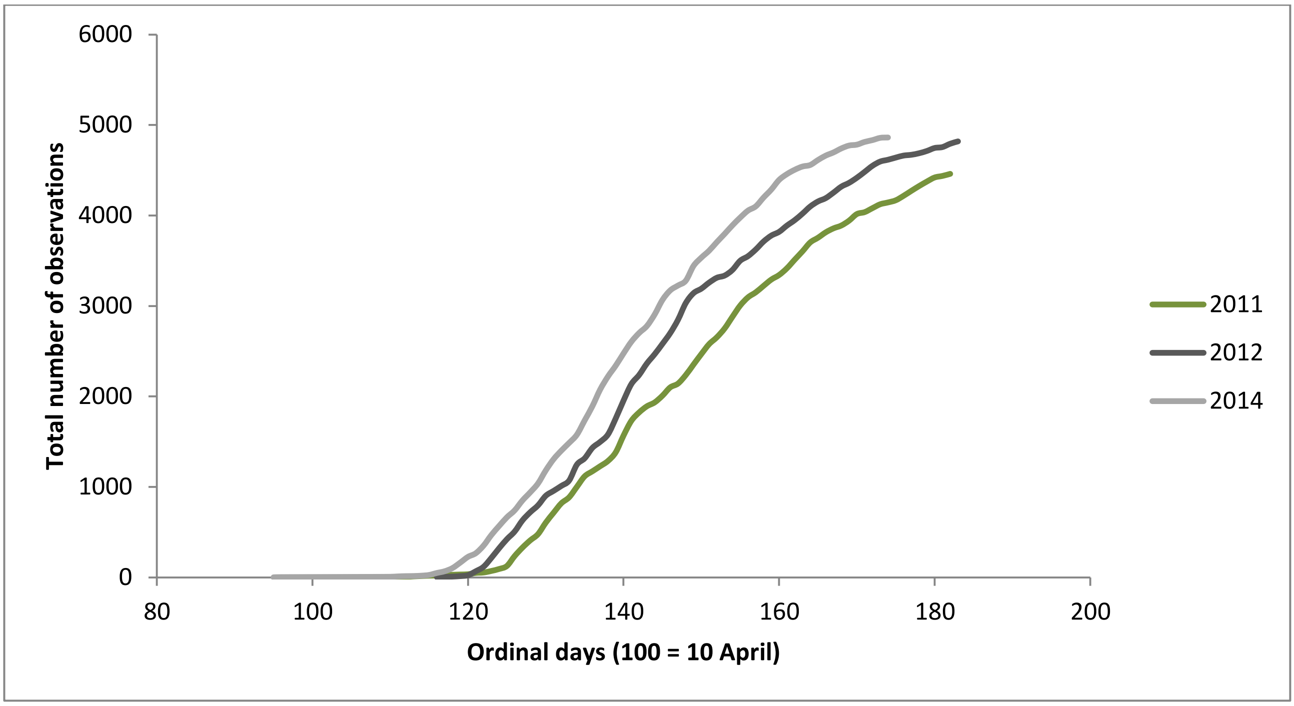

Supplementary Figure 2. Arrival timing of cuckoos in Denmark in 2011, 2012 and 2014. The three curves show the cumulative number of cuckoo observations in Denmark, in the three years with breeding arrival of tracked birds (source: DOFbasen, www.dofbasen.dk, Dansk Ornitologisk Forening

BirdLife Denmark. Accessed 2014 June 23).

Supplementary Movie 1.

Tracking of 11 common cuckoos experimentally displaced from Denmark to southern Spain (red) compared to the migration of non-displaced common cuckoos from Denmark and southern Sweden (grey). Time is shown in the top right corner of the map. Background is a dynamic layer of Normalized difference vegetation index, obtained through the online Data Pool at the NASA Land Processes Distributed Active Archive Center (LP DAAC), USGS/Earth Resources Observation and

Science (EROS) Center, Sioux Falls, South Dakota (https://lpdaac.usgs.gov/data_access). The Movie is created by M. C. Berger, Schäuffelhut Berger GmbH, Firetail Visualization Suite.
